# Childhood vision impairment and blindness in West Africa: public health measures and implications for systemic health

**DOI:** 10.3389/fmed.2024.1349093

**Published:** 2024-02-19

**Authors:** Caleb Yeh, Crystal Huang, Ye Huang, Caleb D. Hartley, Tolulope Fashina, Nathaniel Ashby, Chase Miller, Jessica G. Shantha, Grant A. Justin, R. V. Paul Chan, John G. Mattia, Matthew J. Vandy, Lloyd Harrison-Williams, Jalikatu Mustapha, Jean-Claude Mwanza, Steven Yeh

**Affiliations:** ^1^Truhlsen Eye Institute, University of Nebraska Medical Center, Omaha, NE, United States; ^2^Department of Ophthalmology and Visual Sciences, University of Illinois Chicago, Chicago, IL, United States; ^3^Creighton University School of Medicine, Omaha, NE, United States; ^4^F.I. Proctor Foundation, Department of Ophthalmology, University of California, San Francisco, San Francisco, CA, United States; ^5^Walter Reed Army National Military Medical Center, Bethesda, MD, United States; ^6^National Eye Health Programme, Sierra Leone Ministry of Health and Sanitation, Freetown, Sierra Leone; ^7^Department of Ophthalmology, University of North Carolina, Chapel Hill, NC, United States; ^8^Global Center for Health Security, University of Nebraska Medical Center, Omaha, NE, United States

**Keywords:** childhood blindness, severe vision impairment, measles, vitamin A deficiency, West Africa, refractive error, Ebola, childhood vision impairment

## Abstract

Childhood blindness is an issue of global health impact, affecting approximately 2 million children worldwide. Vision 2020 and the United Nations Sustainable Development Goals previously identified childhood blindness as a key issue in the twentieth century, and while public health measures are underway, the precise etiologies and management require ongoing investigation and care, particularly within resource-limited settings such as sub-Saharan Africa. We systematically reviewed the literature on childhood blindness in West Africa to identify the anatomic classification and etiologies, particularly those causes of childhood blindness with systemic health implications. Treatable causes included cataract, refractive error, and corneal disease. Systemic etiologies identified included measles, rubella, vitamin A deficiency, and Ebola virus disease. While prior public health measures including vitamin A supplementation and vaccination programs have been deployed in most countries with reported data, multiple studies reported preventable or reversible etiologies of blindness and vision impairment. Ongoing research is necessary to standardize reporting for anatomies and/or etiologies of childhood blindness to determine the necessity of further development and implementation of public health measures that would ameliorate childhood blindness and vision impairment.

## 1 Introduction

Childhood vision health, which includes both the prevention and treatment of ophthalmic disease, remains a key global health initiative related to the United Nations Sustainable Development Goals and in the International Agency for the Prevention of Blindness (IAPB) Vision 2020 plan (1). The recent “2030 in Sight” strategy document published by the IAPB has reinforced childhood vision initiatives including the goal of eye health programs in every school by 2030 (2).

The magnitude of childhood blindness is profound, with an estimated 2 million blind children worldwide (3). Blind children also have a higher mortality rate compared to sighted children, given systemic risk factors including measles and vitamin A deficiency that can be associated with mortality and childhood blindness (4). Moreover, the number of blind children living in developing countries within sub-Saharan Africa is disproportionately high. This disparity is due to a combination of factors including supply chain challenges, limited health infrastructure, access to trained subspecialists, and cultural and social factors that may change healthcare-seeking behavior (5). Immunization and nutritional supplementation, which are critical to childhood vision health, for conditions such as measles and vitamin A deficiency (VAD), respectively, may vary between and within countries, although efforts have been made to improve these areas of health prevention. The UNICEF Multiple Indicator Cluster Survey (MICS) has provided valuable insight into country-level data that highlights improvements in vitamin A supplementation and measles vaccination over time (6).

The relationship between childhood blindness and systemic disease may involve infectious disease processes, nutritional deficiencies, and inherited conditions where vision impairment and systemic illness may intersect. Prior research has identified measles and corneal blindness along with VAD as key risk factors (7), although public measures in some countries, including measles immunization and vitamin A supplementation programs, have been deployed to combat these ailments (8). Nonetheless, epidemiologic studies of childhood blindness may reveal additional infectious disease conditions, including rubella, toxoplasmosis, and recent reports of Ebola virus disease (EVD) in association with childhood vision loss.

Within West Africa and other developing countries, childhood blindness surveys have been employed to provide useful information about the epidemiology of blindness (9). We sought to systematically review ophthalmologic studies to determine anatomic locations of blindness, precise etiologies of blindness identified, and whether key relationships between childhood vision impairment and systemic illness could be identified. This synthesis of the literature examines studies of schools for the blind and key informant interviews of communities in multiple countries in West Africa to ascertain the risk factors, anatomic and etiologic causes of childhood vision impairment, as well as systemic disease associations.

## 2 Methods

**Figure 1:**
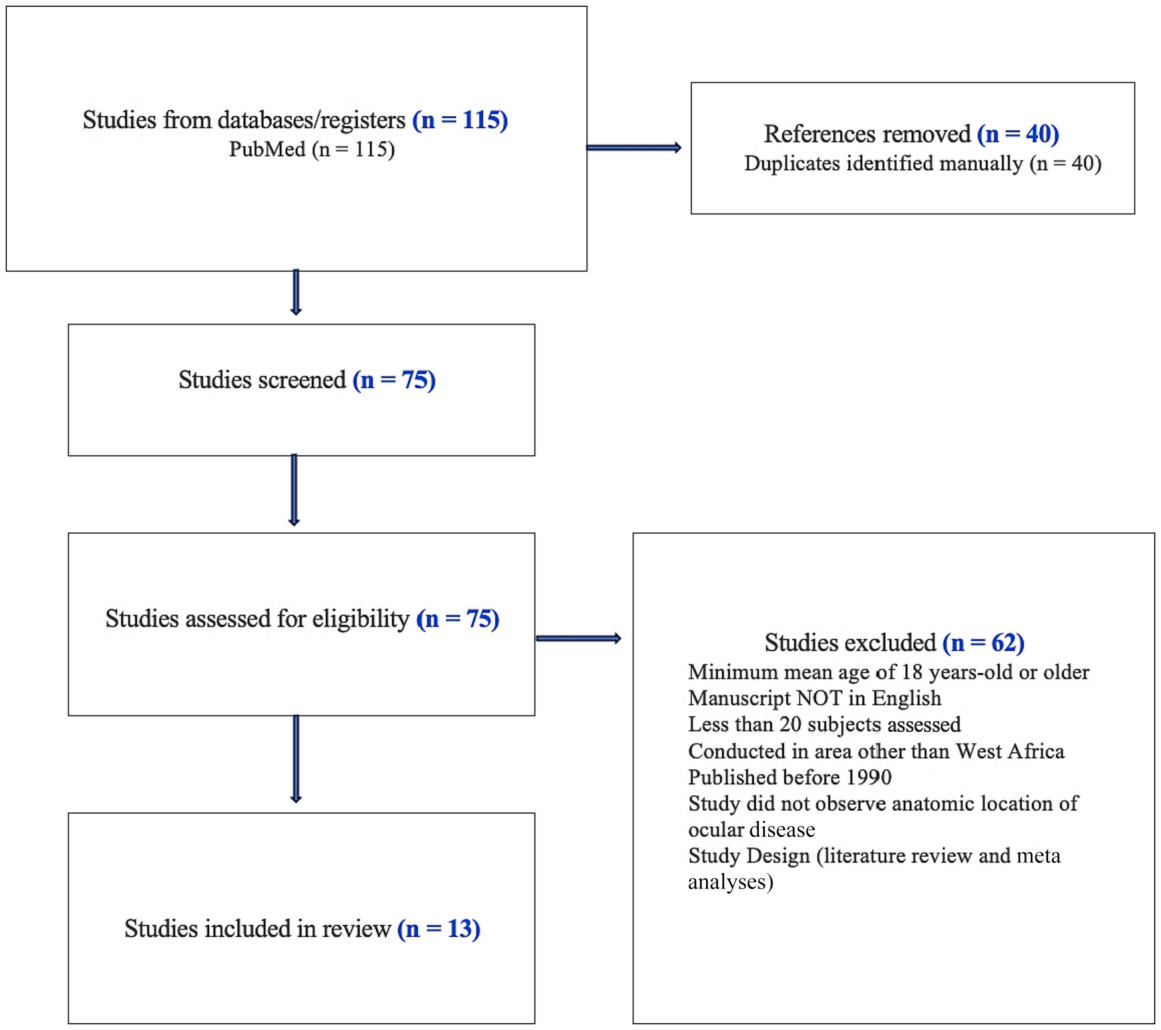
PRISMA diagram.

We performed a PubMed literature search without date restrictions to identify the causes of childhood visual impairment and blindness (VI/BL) in West Africa (Figure 1). We identified a total of 75 papers following the United Nations definition of West Africa and included the following countries: Benin, Burkina Faso, Cabo Verde, Chad, Gambia, Ghana, Guinea, Guinea-Bissau, Ivory Coast, Liberia, Mali, Mauritania, Niger, Nigeria, Saint Helena, Ascension, and Tristan da Cunha, Senegal, Sierra Leone. One study combined outcomes from Togo, Ghana, Benin, and Chile as well as Southeast Asia (10).

Search terms included multiple countries in West Africa, including the previously mentioned United Nations country list. Search terms related to childhood vision impairment included “blindness,” “vision impairment,” and “vision loss.” The majority of articles reviewed followed the WHO definition of vision impairment and blindness for classification of subjects assessed. WHO criteria for “moderate visual impairment” included better eyes worse than 6/18 and equal to or better than 6/60, while “severe visual impairment” included better eyes worse than 6/60 and equal to or better than 3/60. Blindness was defined as worse than 3/60 in the better seeing eye. The articles assessed in our study utilized the WHO/Prevention of Blindness Examination record for children (11). Articles selected for full review met the following criteria: documented visual acuities and reported causes of VI/BL, greater than or equal to 20 subjects assessed, and data were derived from a country in West Africa. Manuscripts could also include research where multiple countries were assessed, provided that at least one West African country was reviewed. Demographic restrictions including maximum mean age of 18 years-old or younger, and studies could include females, males, or unreported. Our literature search focused on peer-reviewed manuscripts in English and studies including quantitative data while excluding systematic reviews and meta-analyses.

Papers selected for a full review were assessed for the following parameters where available: reporting country, practice setting, data extraction method, number of patients assessed, rural or urban setting, associated systemic diseases, study population (e.g., blind school or community-based study), classification of vision loss, year(s) when the study was published, key recommendations, and etiology of disease associated with vision loss. In addition to etiologic causes of blindness, we also extracted the anatomical locations of blindness from the studies. If the study contained both anatomic and etiologic disease associations, both were included in the summary (Table 1) and key observations were summarized in this review.

**Table 1:** Anatomic and etiologic causes of childhood blindness by West African country.

References	Setting/Data extraction method	No. patients assessed	Anatomic diagnosis	Etiologic diagnosis	Associated systemic diagnoses
Nigeria					
Ezegwui et al. (12)	Three schools for the blind in southeastern Nigeria (Oji River, Enugu State; Isulo, Anambra State; and Afara, Umuahia Abia State)	142	Cataract (23.6%) Corneal scarring (21.4%) Phthisis bulbi (12.1%) Glaucoma/Buphthalmos (9.3%) Microphthalmos or Disorganized globe (9.3%) Retina (7.9%) Optic nerve atrophy (7.1%) Uncorrected aphakia (5%) Retinitis pigmentosa (5%)	Measles (25%) Rubella (7.9%) Trauma. (7.2%) HTEM (7.2%)	Measles (25%) Rubella (7.9%)
Mosuro et al. (13)	All the schools for the blind in Oyo State, Nigeria (located in Ogbomosho, Oyo, Ibadan, and Eruwa)	86	Cornea (29.1%) Lens (26.7%) Retina/Optic nerve (18.6%) Optic atrophy (14%) Glaucoma (13%) Globe (10.5%) Cortical blindness (2.3%) Ocular inflammation (3.5%)	Measles (29.1%) Congenital cataract (26.7%) Glaucoma (12.8%) Congenital rubella syndrome (4.7%)	Measles keratopathy/VAD in (29.1%) Rubella (4.7%)
Omolase et al. (14)	Owo School for the Blind	62	Cataract (21%) Glaucoma (12.9%) Phthisis bulbi (9.7%) Microphthalmia (3.2%) Leucoma (9.7%) Retinitis pigmentosa (11.3%) Optic atrophy (12.9%) Squint (3.2%) Aphakia (3.2%) Anophthalmus (1.6%) Maculopathy (1.6%) Pseudophakia (1.6%)	Hereditary (21%) Intrauterine (37.1%) Childhood (16.1%) Unknown (22.6%) Trauma (3.2%)	Toxoplasmosis (6.5%)
Muhammad et al. (15)	Gwadabawa local government area (LGA)/KI Method	58	Corneal opacity (55%) PB (20%) Cataract (15%) Anterior uveitis (10%)	Childhood factor (75%) Hereditary (10%) Couching (5%)	Childhood factors (VAD, infectious keratitis, and trauma) (75%)
Duke et al. (16)	Child Eye health tertiary facility (CEHTF) at the University of Calabar Teaching Hospital using KI Method	1,020	Lens (35%) Cataract (28%) Whole globe (19%) Cornea (16%) Retina (8%) Optic nerve (8%) Refractive error (8%) Cortical blindness (4%) Nystagmus (1%)		NR
Aghaji et al. (17)	3 schools in Southeast Nigeria (data extraction method not specified)	127	Cataract (28.2%) Whole globe (27.6%) Cornea (20.5%) Glaucoma/Bupthalmos (1%) Optic NERVE (7.9%) Retina (3.9%)	Measles/VAD (18.6%) Rubella (5.8%) HTEM (4.8%) Trauma (3.2%)	Measles/VAD (18.6%) Rubella (5.8%)
Olowoyeye et al. (8)	Nigeria Society for the Blind Vocational Training Center, PSB, and Nigeria Farm Craft Center for the Blind	116	Phthisis bulbi (17.2%) Optic nerve atrophy (17.2%) Corneal scarring (12%) Retina dystrophy (7.8%) Cataract (6.9%) Microphthalmos (5.2%) Buphthalmos (5.2%) Retinal detachment (4.3%) Glaucoma (1.7%) Aphakia (3.4%) ROP (0.9%)	Measles (12.9%) CNS infections (8.6%) Rubella (4.3%) Trauma (1.7%) Malaria (1.7%)	Measles (12.9%)
Aghaji et al. (18)	South-Eastern Nigeria (ages 0–15 years)/KI Method	68	Cataract (40%) Optic atrophy (13.3%) Corneal scar (13.3%) Refractive error (13.3%) Microphthalmia (6.7%). Optic nerve hypoplasia (6.7%) Phthisis bulbi (6.7%)	Hereditary factors (26.7%) Prenatal factors (6.7%)	NR
Ghana					
Ntim-Amponsah et al. (19)	School for the Blind at Akropong, Ghana	199	Cornea (42.21%) Congenital and acquired globe abnormalities (37.19%) Lens (23.12%) Glaucoma (15.58%) Retina (9.05%) Optic nerve (8.54%) Tumor (2.01%) Anophthalmos (0.50%)	Measles (26.6%) Traditional Medicine (25.1%) Trauma (3.0%) Malnutrition (2.5%)	Measles (26.6%) Malnutrition (2.5%)
Huh et al. (7)	Wa Methodist School for the Blind in Northern Ghana, survey	190	Optic nerve atrophy (12%) Corneal scar (11%) Glaucoma (9%) Microphthalmos (9%) Cataract (9%) Cortical blindness (7%) Phthisis bulbi (4%) Uveitis (4%) Retinitis pigmentosa (2%) Refractive error (2%)	Other unknown etiology (22%) Abnormality since birth (17%) Hereditary Disease (15%) Measles (10%)	Measles (10%) and Rubella (2%)
Ilechie et al. (20)	The Akropong (the oldest school for the blind in Ghana) and Cape Coast Schools for the blind in southern Ghana, and the Wa School for the Blind in northern Ghana survey	252	Cataract (24.2%) Corneal scarring (19.8%) Glaucoma (9.9%) Corneal dystrophy (5.6%) Microphthalmos (5.2%) Other retinal lesions (5.2%) Cortical blindness (4%) Aphakia (1.6%)	VAD (6.7%) Measles (6.4%) Toxoplasmosis (3.6%) Rubella (2.8%) Trauma (3.6%) HTEM (3.2%) Retinitis pigmentosa (1.2%) Albinism (0.4%) Ophthalmia neonatorum (0.4%) Retinoblastoma (0.4%)	Measles/VAD (13.1%) Rubella (2.8%)
Sierra Leone					
Shantha et al. (21)	Lowell and Ruth Gess Kissy Eye Hospital in Freetown, Sierra Leone	81	Vernal keratoconjunctivitis (21%) Corneal scar (12%) Glaucoma (6%) Uveitis (7%) Synechiae (3%) Cataracts (2%) Corneal edema (2%)		Ebola survivors (70%) Close contact (57%)
Ghana, Togo, Benin					
Gilbert et al. (10)	Blind Schools (does not specify)	284	CS/PB (35.9%) Retina (20.4%) Lens (15.5%) Glaucoma (13%) Whole globe (8.5%) Optic nerve (5.6%) Uvea (1.1%)	VAD/measles/TEM (29%) Rubella (7%) Ophthalmia neonatorum (2%)	VAD/Measles/TEM (29%) Rubella (7%)

HTEM, harmful traditional eye medicine; TEM, traditional eye medicine; ROP, retinopathy of prematurity; CS, corneal scar; PB, phthisis bulbi; VAD, vitamin A deficiency; ROP, retinopathy of prematurity. NR: not reported.

Besides these parameters related to childhood blindness, we also recorded the methodology for each study, and obtained anatomic and etiologic data by distributing surveys to patients using one of two methodologies: (1) School for the blind surveillance methodology; or (2) Key informant (KI) method. The schools for the blind surveillance method involves the selection of these school(s) from the region the study assessed, followed by measurements of the students’ visual acuities and eye examinations. The methodologies often utilized a magnifying loupe and penlight to observe the anterior segment and either the indirect or direct ophthalmoscopes to observe the posterior segment of the eye. The KI method involved the selection and training of KIs to identify children in their communities who are suspected of having visual impairment. The children then received an eye exam, in which their visual acuity, etiology, and anatomical cause of VI/BL were documented.

## 3 Results/findings

Our review of childhood blindness in West Africa resulted in 75 studies of which 13 were selected and abstracted for full review by the two primary authors (CY and CH). The majority of the studies were performed in Nigeria (*n* = 8) with additional studies in Ghana (*n* = 3), Sierra Leone (*n* = 1), and one study that contained data from Togo, Ghana, and Benin (11). The results of our study are shown in Table 1 and are categorized by country.

Patient cohorts were examined from schools for the blind surveillance (*n* = 10) and through KI methods (*n* = 3). Of the 13 studies reviewed, 4 studies (30.8%) classified causes of childhood blindness by anatomic location only and 9 (69.2%) classified studies according to both anatomic location and etiology. We identified 10 of 13 studies (76.9%) where any systemic disease association (i.e., 1 or more attributable systemic conditions) was described in association with childhood blindness.

### 3.1 Etiologic categories of systemic disease associated with childhood vision impairment infectious diseases

A spectrum of pathogens was identified as causes or in association with blindness including viral, parasitic, and bacterial causes. Viral diseases included measles, rubella, and Ebola. Parasitic diseases included toxoplasmosis. Onchocerciasis was not recorded in the manuscripts reviewed. Ophthalmia neonatorum due to chlamydia was also observed.

Measles was the most commonly occurring infectious disease identified in the studies reviewed, appearing as an etiologic cause of severe VI/BL in 9 of 13 (69.2%) studies. Of these studies, the percentage of measles defined as an etiologic cause of severe VI/BL ranged from 10 to 26%. Measles was the most identified etiologic cause of severe VI/BL in 4 (44.4%) studies; however, oftentimes, it was grouped with other causes, including 2 studies in which the collective group including measles was the most common etiologic cause of blindness.

Rubella was the second most common systemic disease defined as an etiologic cause, identified in 6 of 13 studies (46.2%) with percentages ranging from 2 to 7%.

In one study where pediatric Ebola survivors and close-contacts were examined, uveitis was observed in 10.8% of Ebola survivors compared to 1.7% of close-contacts (21). In patients in whom uveitis was identified, vision impairment was observed in a high proportion of individuals. Ocular complications were also found to be significantly associated with a reduction in vision-related quality of life in both Ebola survivors and close-contact control patients.

Non-viral infectious diseases associated with childhood blindness were found in a lower percentage of patients. Specifically, toxoplasmosis varied between 1 and 6.5% while ophthalmia neonatorum was attributed to childhood blindness in 0.4–2% of patients in the studies reviewed.

### 3.2 Nutritional deficiencies

Prior studies have documented the association of VAD with xerophthalmia, corneal ulceration, and blindness. Concomitant measles infection with associated keratitis compounds VAD leading to corneal scarring (CS). In addition, malnourishment and immune dysfunction from VAD predisposes children to measles.

In the setting of severe ulceration and perforation from VAD, phthisis bulbi (PB) may also ensue. Given the inter-relatedness of these conditions, several studies combined VAD and measles as a collective cause of vision loss (13, 17, 19, 20). The proportion of children with blindness due to this combination of VAD and measles ranged between 13.1 and 29% in these studies.

### 3.3 Harmful traditional eye medicine/trauma

Harmful traditional eye medicine (HTEM) is a common cause of severe VI/BL resulting from a combination of factors that may include a lack of access to medical treatment and healthcare services or dissatisfaction with health care providers. These treatments can involve the direct instillation of herbal preparations into the eye (19). HTEM was found to be the etiological cause of blindness in four studies, ranging from 4.8% to as high as 25.1% of patients assessed.

Trauma was found to be an etiological cause of blindness in six studies, varying between 2.3 and 7%. Whether other systemic traumatic injuries were observed concomitantly was not specifically recorded.

### 3.4 Hereditary and genetic causes

Hereditary factors classified as an etiologic cause of severe VI/BL were found to be the etiologic cause in three studies ranging from 15 to 26.7%.

### 3.5 Anatomic locations of disease leading to childhood vision impairment

Besides assessing the systemic associations of childhood blindness, multiple studies classified childhood blindness according to anatomic location including corneal scarring/phthisis bulbi (CS/PB), cataract, or glaucoma. The most common anatomic locations defined are summarized.

### 3.6 Cornea

Corneal disease was the most commonly defined anatomic location of blindness observed in our review of the literature. In four studies, corneal disease was the most common anatomic cause or location of blindness, reported in 29.1–42% of patients (10, 13, 15, 19). The most common cause documented was CS, and often associated with PB, leading to these two terms appearing as the cause of blindness in several studies. The combined category of CS and PB was responsible for 35.9% of visual impairment in a study by Gilbert et al. (10). Corneal opacity, which potentially could be associated with CS, was observed in 55% of the patients with visual impairment in a report by Muhammad et al. (15). In this report, CS alone was the most common cause of blindness. Seventy-five percent of etiologies in this report were associated with a childhood factor including VAD and measles, comprising patients with CS and PB.

### 3.7 Lens

Of the 13 studies reviewed, the most common lenticular conditions leading to blindness—“cataract” and “aphakia”—were present in 11 and 5 studies, respectively. Cataract was observed to be an anatomic cause of blindness in 11 studies, leading to blindness from an estimated 7–40% of the VI/BL and notably was the leading cause of blindness in 5 studies (12, 14, 17, 18, 20). Patients were referred for surgery in several reports, but the outcomes were not reported in these manuscripts.

Aphakia was associated with 1.6–5% of VI/BL. Aphakia was deemed to be associated with rubella or intrauterine causes of blindness in the studies where aphakia was documented.

### 3.8 Retina

Blindness due to retinal disease was variable across 5 studies in which these anatomic locations were documented and ranged from 3.9 to 20.4%. These diseases included retinoblastoma, retinitis pigmentosa (RP), retinopathy of prematurity (ROP), and retinal detachment. RP, a hereditary disease, was the most commonly appearing retinal cause of blindness while retinoblastoma and ROP were documented in <1% of cases in two independent studies (8, 20). Of the 4 studies discussing RP, the percentage of blindness caused by RP ranged from 1.2 to 11.3%. Given the findings that may overlap between retinal disorders and optic nerve disease, one study combined both anatomic locations and reported that 18.6% of patient blindness was located at the retina and optic nerve (13).

### 3.9 Optic nerve and glaucoma

The most common optic nerve finding associated with blindness was optic atrophy, which was reported in 6 studies with a frequency of blindness association ranging between 7.1 and 17.2%. Glaucoma was reported in 11 studies and led to VI/BL in 2 to 40% of patients with vision impairment. Congenital glaucoma, which was sometimes grouped with buphthalmos, was identified in over 12% of patients in one study (17).

### 3.10 Whole globe and other categories of childhood blindness

The terminology “Whole Globe” was included as an anatomic location or cause of blindness in all the studies reviewed. This category was perhaps the most diverse group, encompassing conditions such as glaucoma and buphthalmos, as well as conditions such as PB. PB was described in association with corneal scarring in multiple studies, representing an unfortunate, final common pathway from multiple etiologies (e.g., untreated uveitis, corneal perforation from measles/VAD and use of HTEM).

Microphthalmia/Microphthalmos also fell into this category as a cause of visual impairment in 5 studies. This disease could also be attributed to congenital rubella and while infrequent, the range of blindness associated with microphthalmos varied in a significant minority of patients ranging from 3.2 to 9%.

Other notable categories of childhood blindness that were observed infrequently included refractive error (2–13%) and uveitis (4–10%) including patients with a history of EVD or clinical manifestations indicative of toxoplasmosis. Cortical blindness was identified ranged from 2.3 to 7% in 4 studies where this was reported.

### 3.11 Child vision health and quality-of-life

The relationship of child vision health and impairment with quality-of-life reduction has been reported previously. However, whether pediatric vision health predicts poorer quality-of-life in resource-limited countries is less well-characterized. In the literature search conducted, we also assessed whether specific relationships were observed between child VI/BL and quality-of-life metrics.

Shantha et al. assessed vision impairment in a cross-sectional study of EVD survivors or close-contact control patients at the Lowell and Ruth Gess Eye Hospital in Sierra Leone (21). Eighty-one pediatric patients were evaluated for visual acuity, eye disease, and quality-of-life changes. Quality of Life was assessed using the Pediatric Quality of Life Inventory Version 4·0 (PedsQL) and Effect of Youngsters Eyesight on Quality-of-Life (Eye-Q), with a comparison between Ebola survivors and close contact control patients. A high proportion of EVD survivors showed eye disease with 47% of eyes demonstrating ocular disease, while 32% of eyes in the close-contact control population also showed ocular findings. Interestingly, when ocular findings were observed, individuals in both the EVD and control cohort showed a reduction in vision-related quality of life and function. These indicate that while pediatric EVD survivors were observed to have a higher prevalence of uveitis and ocular inflammation, the eye disease burden within the entire cohort including control patients was high and contributed to reduced quality of life.

## 4 Impact of public health interventions on child vision health and blindness

While the studies in West Africa are illustrative of the burden of disease ranging from anterior segment to posterior segment disease, as well as whole globe changes, public health interventions and their impact have continued to be deployed with variability across countries and even regions within countries. Within Nigeria, Olowoyeye et al. described a significant decrease in VI/BL from 2000 to 2017, which could be associated with public health measures including an increase in measles immunization, vitamin A supplementation, and the establishment of tertiary eye facilities through implementation initiatives aligned with the WHO’s VISION 2020 initiative (8).

In Ghana, health sector reform in 1997 coincided with an increase in measles immunization coverage and a notable decrease in the proportion of CS/PB-caused VI/BL (7). Within Ilechie et al. reported a higher proportion of students located in South Ghana became blind between the age cohort of 1–15 compared to North Ghana (20). This observation could potentially be associated with an increase in the coverage of measles immunization and vitamin A supplementation programs within Northern Ghana. Moreover, the use of HTEM remains a leading issue, as Ntim-Amponsah et al. reported corneal blindness due to HTEM as around 13% people who had eye disease in a Ghanaian community resorted to traditional herbal medicine (19). Evidenced-based, public health measures targeting both different countries and regions within countries are clearly needed.

## 5 Discussion

A comprehensive synthesis of the literature from West Africa showed that the major anatomic causes of blindness included CS and lens/cataract-related, while a significant minority of patients showed evidence of VI/BL from optic neuropathy, glaucoma, and retinal disease. Within the literature reviewed, viral diseases including measles, rubella, and Ebola virus were observed to lead to vision impairment, but nutritional deficiencies, in particular, VAD contributed to disease morbidity (Table 2). Other genetic/hereditary conditions and neoplastic disease including retinoblastoma were observed. However, many of the most prevalent diseases and etiologies for childhood blindness are preventable including cataract, refractive error, and corneal conditions not associated with PB. In addition, with growing interventions and the importance of childhood screening, blindness secondary to congenital glaucoma and retinal disease can also be prevented with early identification and prompt treatment.

**Table 2:** Leading causes of childhood blindness in West Africa.

Category	Key causes
Infectious diseases	Measles Rubella Malaria Ebola Other central nervous system infections Ophthalmia neonatorum
Nutritional deficiencies	Vitamin A deficiency (VAD)
Prenatal factors	Congenital rubella syndrome Toxoplasmosis
Hereditary factors	Glaucoma Retinitis pigmentosa
Others	Trauma Harmful traditional eye medicine Couching Cortical blindness

Another major focus of this synthesis of the literature from West Africa was related to the relationships of systemic illness with childhood blindness, given prior documented relationships between vision loss and childhood morbidity and mortality. Vitamin A supplementation has led to widespread reduction in VAD symptoms and associated morbidity and mortality; however, within the literature reviewed, we still found 13.1–29.1% of patients who had ocular findings associated with VAD. Therefore, while the increase in vitamin A supplementation has hinted toward improvements in ocular diseases instigated by VAD, the progress has varied between countries and even within countries. Within Nigeria, improved eye health has been realized. Yet, within Ghana, improvements were seen through a decrease in CS/PB cases in northern Ghana from 1987 to 2014 compared to an increase in cases from 1989 to 2003 within southern Ghana (20). Rubella was the only other systemic disease that was prevalent enough to warrant a specific recommendation, which suggested additional research be conducted to assess the necessity of vaccination in Nigeria (8).

Identification of ophthalmic findings due to malaria, chlamydia and gonorrhea in ophthalmia neonatorum, and Ebola are reminders of the range of infectious diseases that may lead to eye disease, potentially requiring anti-infective or anti-inflammatory therapies.

Our review of the literature revealed some limitations inherent to reporting with the two major methods of reporting being the KI method or the child schools for the blind surveillance method. Potential advantages of utilizing the KI method include avoidance of sampling bias through surveillance of government areas/local communities as opposed to just blind schools. However, because the process is labor intensive, undercoverage bias is possible due to the small number of VI/BL children whom key informants can identify. Additionally, a relatively high proportion of identified individuals do not show up to their eye examinations, further decreasing the number of patients assessed.

Limitations of schools for the blind surveillance method include the high male-to-female ratio, which may potentially reflect selection bias as many female VI/BL students are withheld from attending schools for the blind. In some scenarios, SVI children might attend regular schools or be denied entry into schools for the blind due to other handicaps. Schools for the blind studies are also potentially under-representative of rural areas as all the studies of blind schools reviewed were set in urban areas. National approaches to population-based data in West African countries could minimize the population biases associated with assessment of children from blind schools and could provide more accurate epidemiological data. Significant heterogeneity was also observed in reporting as overlap between anatomic and precise etiologic causes of blindness. For example, congenital cataract could be reported as both an anatomic and etiologic cause of blindness, but the underlying etiology (i.e., infectious, genetic, or other) could not be determined from the manuscript. Despite these limitations, a full understanding of childhood blindness through literature from West Africa and other countries within sub-Saharan Africa represent an opportunity to align terminology, identify relationships between child vision health and systemic illness that would inform public health measures for vision health intervention.

Public health recommendations including VAD and measles immunization were found to have made positive impacts on reporting countries such as Nigeria and Northern Ghana, which are consistent with the United Nations Sustainable Development Goals and WHO’s Vision 2020 plan. The importance of childhood vision screening through school programs is further emphasized in the IAPB 2030 in Sight strategic document. Other key recommendations that have been emphasized include additional research and studies to obtain a better grasp on both regional etiological patterns and the necessity of certain vaccines, eye health education of local communities to raise awareness on the benefits of early screenings of infants to identify diseases like cataract and glaucoma. A better understanding of traditional eye medicines and remedies, as well as awareness of primary and tertiary eye care services could potentially reduce cases of preventable blindness. Interestingly, as there were cases of treatable vision loss identified in children enrolled in schools for the blind, strengthening vision health and vision rehabilitation services for schools for the blind could also be emphasized across public and private sectors.

## 6 Conclusion

With the wide range of causes of childhood blindness reported, the spectrum of disease in different countries within West Africa and landscape of childhood vision impairment likely requires further investigation to inform public health measures. Placing a greater emphasis on the research of etiologic causes of childhood blindness would help initiate preventative measures unique to each region while observing the development and evolution of these causes. Additional research focusing on a national assessment of the relationship between childhood blindness and infectious diseases could also improve the distribution of immunizations such as the MMR vaccine. The implementation of standardized vision screening protocols with national databases could align defined causes of childhood blindness with resources via local Ministries of Health and external partnerships.

## Author contributions

CY: Investigation, Methodology, Resources, Writing – original draft, Writing – review & editing, Data curation, Formal analysis. CH: Data curation, Formal analysis, Investigation, Methodology, Resources, Writing – original draft, Writing – review & editing, Conceptualization. YH: Conceptualization, Investigation, Methodology, Resources, Writing – review & editing, Project administration, Supervision. CDH: Investigation, Methodology, Project administration, Resources, Supervision, Writing – review & editing, Data curation, Formal analysis. TF: Data curation, Investigation, Methodology, Project administration, Resources, Supervision, Writing – review & editing, Funding acquisition. NA: Investigation, Methodology, Project administration, Writing – review & editing, Conceptualization, Visualization. CM: Investigation, Methodology, Project administration, Visualization, Writing – review & editing. JS: Investigation, Methodology, Project administration, Writing – review & editing, Funding acquisition, Resources, Supervision. GJ: Investigation, Methodology, Project administration, Supervision, Writing – review & editing, Conceptualization. RC: Investigation, Methodology, Project administration, Supervision, Writing – review & editing, Resources, Validation, Visualization. JMa: Investigation, Project administration, Supervision, Writing – review & editing. MV: Investigation, Project administration, Supervision, Writing – review & editing. LH-W: Investigation, Project administration, Supervision, Writing – review & editing, Conceptualization, Resources, Validation. JMu: Conceptualization, Investigation, Project administration, Resources, Supervision, Writing – review & editing, Methodology. J-CM: Conceptualization, Investigation, Methodology, Project administration, Resources, Writing – review & editing, Funding acquisition. SY: Conceptualization, Funding acquisition, Investigation, Methodology, Project administration, Resources, Writing – review & editing, Supervision, Writing – original draft.

## References

[1] World Health Organization. Preventing blindness in children: report of WHO/IAPB scientific meeting. Geneva: WHO (2000).

[2] Gilbert C Foster A. Blindness in children: control priorities and research opportunities. Br J Ophthalmol. (2001) 85:1025–7. doi: 10.1136/bjo.85.9.1025, PMID: 11520746 PMC1724126

[3] Bourne R Steinmetz JD Flaxman S Briant PS Taylor HR Resnikoff S. Trends in prevalence of blindness and distance and near vision impairment over 30 years: an analysis for the global burden of disease study. Lancet Glob Health. (2021) 9:e130–43. doi: 10.1016/S2214-109X(20)30425-3, PMID: 33275950 PMC7820390

[4] The International Agency for the Prevention of Blindness. 2030 in sight strategy document. (2021). Available at: https://www.iapb.org/learn/resources/2030-in-sight-strategy-document/.

[5] Bechange S Jolley E Tobi P Mailu E Sentongo J Chulu T. Understanding patient health-seeking behavior to optimize the uptake of cataract surgery in rural Kenya, Zambia, and Uganda: findings from a multisite qualitative study. Int Health. (2022) 14:i57–63. doi: 10.1093/inthealth/ihab061, PMID: 34581785 PMC8986356

[6] 2021 multiple Indicator cluster survey/National Immunization Coverage Survey Report. UNICEF for every child. Available at: www.unicef.org (Accessed January 28, 2024). 10.1093/infdis/jiad476 PMC11272093 39052714

[7] Huh GJ Simon J Prakalapakorn GS. Causes of childhood blindness in Ghana: results from a blind school survey in upper West region, Ghana, and review of the literature. Int Ophthalmol. (2018) 38:1415–23. doi: 10.1007/s10792-017-0600-9, PMID: 28612329 PMC5729053

[8] Olowoyeye AO Musa KO Aribaba OT Onakoya AO Akinsola FB. Pattern of childhood visual impairment and blindness among students in schools for the visually impaired in Lagos state: an update. Niger Postgrad Med J. (2018) 25:105–11. doi: 10.4103/npmj.npmj_27_18, PMID: 30027922

[9] Steinkuller PG Du L Gilbert C Foster A Collins ML Coats DK. Childhood blindness. J Am Assoc Pediatr Ophthalmol Strabismus. (1999) 3:26–32. doi: 10.1016/s1091-8531(99)70091-1 10071898

[10] Gilbert CE Canovas R Hagan M Rao S Foster A. Causes of childhood blindness: results from West Africa, South India and Chile. Eye. (1993) 7:184–8. doi: 10.1038/eye.1993.39 8325414

[11] World Health Organization. International statistical classification of diseases and related health problems, 11th revision (ICD-11): chapter 7 - vision function. Geneva: World Health Organization (2019). Available at: https://iris.who.int/bitstream/handle/10665/328717/9789241516570-eng.pdf?sequence=18 (Accessed December 30, 2023).

[12] Ezegwui IR Umeh RE Ezepue UF. Causes of childhood blindness: results from schools for the blind in south eastern Nigeria. Br J Ophthalmol. (2003) 87:20–3. doi: 10.1136/bjo.87.1.20, PMID: 12488255 PMC1771452

[13] Mosuro AL Ajaiyeoba AI Bekibele CO Eniola MS Adedokun BA. Survey of low vision among students attending schools for the blind in Nigeria: a descriptive and interventional study. Middle East Afr J Ophthalmol. (2012) 19:382–91. doi: 10.4103/0974-9233.102744, PMID: 23248540 PMC3519125

[14] Omolase CO Aina AS Omolase BO Omolade EO. Causes of blindness and visual impairment at the school for the blind Owo, Nigeria. Ann Ibadan Postgrad Med. (2008) 6:49–52. doi: 10.4314/aipm.v6i1.64042, PMID: 25161445 PMC4111018

[15] Muhammad N Nuhu MM Aliyu JM Rabiu MM. Tracing children with blindness and visual impairment using the key informant survey in a district of North-Western Nigeria. Middle East Afr J Ophthalmol. (2010) 17:330–4. doi: 10.4103/0974-9233.71601, PMID: 21180434 PMC2991451

[16] Duke R Otong E Iso M Okorie U Ekwe A Courtright P. Using key informants to estimate prevalence of severe visual impairment and blindness in children in Cross River state, Nigeria. J AAPOS. (2013) 17:381–4. doi: 10.1016/j.jaapos.2013.05.004, PMID: 23911130

[17] Aghaji A Okoye O Bowman R. Causes and emerging trends of childhood blindness: findings from schools for the blind in Southeast Nigeria. Br J Ophthalmol. (2015) 99:727–31. doi: 10.1136/bjophthalmol-2014-305490 25472948

[18] Aghaji AE Ezegwui IR Shiweobi JO Mamah CC Okoloagu MN Onwasigwe EN. Using key informant method to determine the prevalence and causes of childhood blindness in south-eastern Nigeria. Ophthalmic Epidemiol. (2017) 24:401–5. doi: 10.1080/09286586.2017.1320412, PMID: 28532291

[19] Ntim-Amponsah CT Amoaku WMK. Causes of childhood visual impairment and unmet low-vision care in blind school students in Ghana. Int Ophthalmol. (2008) 28:317–23. doi: 10.1007/s10792-007-9134-x, PMID: 17898940

[20] Ilechie A Wanye S Abraham CH Sarpong JB Abu E Abokyi S. Inter-regional trends in causes of childhood blindness and low vision in Ghana. Clin Exp Optom. (2020) 103:684–92. doi: 10.1111/cxo.13041 31916287

[21] Shantha JG Canady D Hartley C Cassedy A Miller C Angeles-Han ST. Ophthalmic sequelae and psychosocial impact in pediatric Ebola survivors. eClinicalMedicine. (2022) 49:101483–3. doi: 10.1016/j.eclinm.2022.101483, PMID: 35747182 PMC9167858

